# Effect of Expansion of Abbreviations and Acronyms on Patient Comprehension of Their Health Records: A Randomized Clinical Trial

**DOI:** 10.1001/jamanetworkopen.2022.12320

**Published:** 2022-05-13

**Authors:** Lisa Grossman Liu, David Russell, Meghan Reading Turchioe, Annie C. Myers, David K. Vawdrey, Ruth M. Masterson Creber

**Affiliations:** 1 Department of Biomedical Informatics, Columbia University, New York, New York; 2 Department of Sociology, Appalachian State University, Boone, North Carolina; 3 Department of Population Health Sciences, Division of Health Informatics, Weill Cornell Medicine, New York, New York; 4 Geisinger, Danville, Pennsylvania

## Abstract

This randomized clinical trial compares patient comprehension of electronic health records in the US when common medical abbreviations are expanded.

## Introduction

In 2020, an estimated 100 million US residents accessed their health records online.^1^ That number likely increased beginning April 2021, when US federal rules implemented the 21st Century Cures Act requiring electronic health information to be made freely accessible.^2^ Unfortunately, medical abbreviations and acronyms often limit patient understanding of health records. Automated expansion is one potential solution; however, it is unclear whether the magnitude of its effect is sufficiently large to justify costly implementation at scale. In this prospective, 2-arm, parallel, individually randomized clinical trial at 3 US hospitals in metropolitan areas, we estimated how much expansion increases patient comprehension of 10 common abbreviations in their health records.

## Methods

This trial used a purposive sample representative on age, gender, and race that was enrolled between February 2020 and August 2021 (ClinicalTrials.gov NCT05297942) (trial protocol and statistical analysis plan in Supplement 1). To isolate the main effect, we included only English-speaking adult patients with diagnosed heart failure. The Weill Cornell Medicine institutional review board approved the study and all participants provided written informed consent. This trial followed the Consolidated Standards of Reporting Trials Extension (CONSORT Extension) reporting guideline (eFigure in Supplement 2).

Participants were individually randomized to read clinical text with abbreviations (control group, 30 participants) or with expansions (intervention group, 30 participants). The abbreviations and expansions included were *hrs* (hours), *MD* (medical doctor), *BP* (blood pressure), *ED* (emergency department), *yo* (year old), *pt* (patient), *HF* (heart failure), *hx* (history), *HTN* (hypertension), and *MI* (myocardial infarction). We included abbreviations and expansions of varied difficulty as rated by clinicians (data available upon request).

The primary outcome was overall comprehension, assessed using the International Organization for Standardization Method for Testing Comprehension (ISO 9186),^3,4^ and defined as the summary (ie, count) score of the total number of abbreviated or expanded terms comprehended. Baseline demographics, socioeconomic characteristics, and health literacy scores^5^ were collected. Race and ethnicity was self-reported and collected for purposive sampling. For the primary outcome, *P* < .05 in 2-sided tests was considered significant.

## Results

Sixty randomized patients (mean [SD] age, 66 [16] years; 18 [30%] women) completed the trial and were included in the analysis (Table 1). Overall comprehension scores were significantly greater among patients in the intervention group who received expansions than among patients in the control group who received abbreviations (95% vs 62%, respectively; comprehension score, 9.5/10 [95% CI, 9.3 to 9.7] vs 6.2/10 [95% CI, 5.3 to 7.0]; *P* < .001).

**Table 1.** Participant Characteristics

Variable	Participants, No. (%)		
	Overall (N = 60)	By group	
		Abbreviations (n = 30)	Expansions (n = 30)
Demographics			
Age, mean (SD), y	66 (16)	67 (15)	65 (18)
Gender			
Men	42 (70)	22 (73)	20 (67)
Women	18 (30)	8 (27)	10 (33)
Nonbinary	0	0	0
Race			
Asian	2 (3)	2 (7)	0
Black	12 (20)	3 (10)	9 (30)
White	41 (68)	21 (70)	20 (67)
Mixed	1 (2)	1 (3)	0
Native American	1 (2)	1 (3)	0
Prefer not to answer	3 (5)	2 (7)	1 (3)
Ethnicity			
Hispanic/Latinx	7 (12)	6 (20)	1 (3)
Not Hispanic/Latinx	48 (80)	20 (67)	28 (93)
Prefer not to answer	5 (8)	4 (13)	1 (3)
Socioeconomic status			
Education			
High school graduate or less	11 (18)	5 (17)	6 (20)
Some college or associate’s degree	13 (22)	7 (23)	6 (20)
Bachelor’s degree	18 (30)	12 (40)	6 (20)
Graduate degree	18 (30)	6 (20)	12 (40)
Financial resources^a^			
Not enough	17 (28)	6 (20)	11 (37)
Enough	31 (52)	17 (57)	14 (47)
More than enough	12 (20)	7 (23)	5 (17)
Insurance status			
Employer-sponsored	13 (22)	6 (20)	7 (23)
Public (Medicare or Medicaid)	45 (75)	23 (77)	22 (73)
Self-purchased	1 (2)	1 (3)	0
Other	1 (2)	0	1 (3)
Disability status			
Physical disability	13 (22)	6 (20)	7 (23)
Vision or hearing disability	7 (12)	3 (10)	4 (13)
Other disability	14 (23)	9 (30)	5 (17)
No disability	26 (43)	12 (40)	14 (47)
Health literacy			
Health literacy score			
Adequate	35 (58)	16 (53)	19 (63)
Inadequate	25 (42)	14 (47)	11 (37)

^a^ Self-reported selection.

Significant differences in comprehension were observed only for moderately difficult terms such as HTN (hypertension) (Table 2). In a subgroup analysis of control participants, only inadequate health literacy was significantly associated with comprehension of fewer abbreviations (adjusted risk ratio, 0.69; 95% CI, 0.49 to 0.98; *P* = .04).

**Table 2.** Comprehension Differences Between Abbreviated and Expanded Terms

Abbreviation	Comprehension, percentage score (95% CI)	Expansion	Comprehension, percentage score (95% CI)	*P* value, adjusted
hrs	93 (84-100)	hours	100 (100-100)	>.99
MD	93 (84-100)	medical doctor	100 (100-100)	>.99
BP	83 (69-97)	blood pressure	100 (100-100)	.23
ED	67 (49-85)	emergency department	100 (100-100)	.007
yo	90 (79-100)	year old	100 (100-100)	.83
pt	67 (49-85)	patient	100 (100-100)	.007
HF	20 (5-35)	heart failure	100 (100-100)	<.001^a^
hx	43 (25-62)	history	100 (100-100)	<.001^a^
HTN	23 (7-39)	hypertension	97 (90-100)	<.001^a^
MI	37 (18-55)	myocardial infarction	53 (34-72)	>.99

^a^ Significant at an adjusted *P* value of .005.

## Discussion

Expansion of 10 common medical abbreviations compared with no expansion significantly increased overall comprehension of the abbreviations from 62% to 95%. These findings suggest that post hoc or automated expansion of medical abbreviations and acronyms can improve patient understanding of their health information, and may benefit ongoing national efforts to provide patients with electronic access to their own documentation.

Even in this population with substantial prior exposure to the health care system, comprehension of common abbreviations such as MI or HTN remained below 40%, much lower than clinicians initially estimated. Clinicians should be mindful that patient comprehension of abbreviations may be much lower than expected.^6^ Additionally, our data suggest that expansion is an efficacious standalone solution only for certain abbreviations and acronyms. Abbreviations with well-understood meanings while abbreviated (eg, hrs, MD) and poorly understood meanings while expanded (eg, myocardial infarction) did not benefit significantly from the intervention.

This study had several limitations. Ethnicity was imbalanced between study groups by chance; however, sensitivity analyses using multivariable models that controlled for study group and ethnicity demonstrated the imbalance did not affect our conclusions. We restricted our sample to 1 disease condition, which was necessary to isolate the main effect. Results may not generalize to other populations.

## Notes

Supplement 1.
Trial Protocol and Statistical Analysis Plan

Supplement 2. **eFigure.** CONSORT Flow Diagram

Supplement 3.
Data Sharing Statement
