# Supplementary material for: Effect of Expansion of Abbreviations and Acronyms on Patient Comprehension of Their Health Records: A Randomized Clinical Trial
Source: JAMA Netw Open. 2022 May 13;5(5):e2212320. doi: 10.1001/jamanetworkopen.2022.12320 (PMC9107024; doi:10.1001/jamanetworkopen.2022.12320)
Supplement: Supplement 1. — Trial Protocol and Statistical Analysis Plan [file jamanetwopen-e2212320-s001.pdf]

Grossman Liu L, Russell D, Reading Turchioe M, Myers AC, Vawdrey DK, Masterson Creber RM. Effect of expansion of abbreviations and acronyms on patient comprehension of their health records: a randomized trial. *JAMA Netw Open.* 2022;5(5):e2212320. doi:10.1001/jamanetworkopen.2022.12320

Trial Protocol and Statistical Analysis Plan

Effect of Expansion of Abbreviations and Acronyms on Patient Comprehension of their Health Records: A Randomized Controlled Trial

Coordinating Investigator

Ruth Masterson Creber, PhD, RN
Department of Population Health Sciences
425 E 61st St, New York, NY , 10065
rmc2009@med.cornell.edu
+1 646 962 2435

Version 3.0

Finalized 28 March 2022

This version is effectively equivalent to the original trial protocol, with small revisions made upon reviewer request to improve clarity for the reader.

IRB Protocol 1804019182

Table of Contents

Abbreviations and Acronyms 3

Synopsis 4

1. Background and Objectives 5
   - 1.1. Background 5
   - 1.2. Long-Term Goal 5
   - 1.3. Study Objectives 6
   - 1.4. Control of Confounding 6

2. Study Design 7
   - 2.1. General 7
   - 2.2. Participants 7
   - 2.3. Baseline Data 7
   - 2.4. Intervention 10
   - 2.5. Randomization 11
   - 2.6. Outcome 11
   - 2.7. Procedures 12
   - 2.8. Adherence to Guidelines 12

3. Statistical Analysis 13
   - 3.1. Data Management 13
   - 3.2. Sample Size Calculation 13
   - 3.3. Main Analysis 13
   - 3.4. Subgroup Analysis 13

4. References 14

Abbreviations and Acronyms

CARD	Clinical Abbreviation Recognition and Disambiguation
CONSORT	Consolidated Standards of Reporting Trials
COVID	Coronavirus Disease
EHR	Electronic Health Record
EMPI	Enterprise-wide Master Patient Index
HIPAA	Health Insurance Portability and Accountability Act of 1996
HPI	History of Present Illness
ISO	International Organization for Standardization
MRN	Medical Record Number
NY	New York
NYHA	New York Heart Association
REDCap	Research Electronic Data Capture
U.S.	United States

Synopsis

Title	Effect of Expansion of Abbreviations and Acronyms on Patient Comprehension of their Health Records: A Randomized Controlled Trial
Keywords	Abbreviations, Acronyms, Personal Health Records, Patient Portals, Consumer Health Informatics, Patient Access to Records
Sponsor	Department of Population Health Sciences, Division of Health Informatics, Weill Cornell Medicine, New York, NY, USA
Sites	1 geographic area, 3 urban hospitals
Schedule	February 2020 – August 2021: Data Collection<br>September – December 2021: Data Analysis<br>January – March 2022: Dissemination
Design	Prospective, two-arm, parallel, individually randomized controlled trial
Objectives	(1) To better characterize the extent of miscommunication due to abbreviations in health records<br>(2) To evaluate the need for potential solutions such as automated abbreviation expansion
Intervention	Study control is abbreviations and acronyms, and study intervention is expansion of abbreviations and acronyms. Patients will be computer-randomized to the control or intervention in a 1:1 ratio.
End Points	The primary end point is objective comprehension, or whether the participant can define the abbreviation or expansion (binary yes or no variable), as judged by a trained recruitment coordinator according to pre-specified definitions. We will not assess any secondary end points.
Sample Size	Our planned sample size of 60 patients will provide 80% power to detect a 33 percentage point difference in our primary outcome at the 5% significance level. This sample size relies on a comprehension rate of 89% for the intervention and 56% for the control group, based on prior predictions by clinician experts.
Analyses	A two-sided unequal variances t-test with a significance threshold of $P<0.05$ will be used to examine for differences in overall comprehension scores between study groups. Fisher's exact test of independence will be used to test for differences in comprehension rates between abbreviated and expanded versions of each term. As a secondary analysis, association of baseline characteristics with abbreviation and acronym comprehension in the control group will be assessed.

1. Background and Objectives

1.1. Background

A decade ago, only 10% of U.S. healthcare organizations used an electronic health record (EHR), whereas more than 99% do today.[1–4] The rapid adoption of EHRs has radically transformed health communication. Previously, health records primarily consisted of providers handwriting notes in paper charts, rarely seen by outsiders. Today, EHRs integrate data, information, and knowledge from dozens of sources, viewable by providers, patients, and caregivers alike. Accordingly, more patients can instantly access their health records online than ever before, and millions of patients accessed their personal health information online last year.[5–8]

Comprehension of personal health information is challenging for most patients,[9–13] but has the potential to prevent medical errors,[14–19] increase shared decision-making,[20–25] and improve health outcomes.[26,27] According to federal policy, hospitals must allow patients to *view, download, and transmit* their own health information.[28,29] As a result, the percentage of healthcare organizations offering online patient portals increased from 43% in 2013 to 92% in 2015.[30,31] This transparency has been hailed as the next "blockbuster drug" and "healthcare revolution." Arguably, one promising form of transparency is giving patients their doctors' notes. Over 22 million U.S. individuals accessed their doctors' notes in early 2018, a year-over-year increase of more than 120%.[32] Access to notes helps patients take medications as prescribed, be better prepared for future visits, and better understand their illness conditions.[5,33–39]

Unfortunately, tools to help patients *comprehend, interpret, and use* their health information have not evolved concomitantly.[40–43] In our previous research, we determined that clinical abbreviations and acronyms (hereby "abbreviations") present a major barrier to patient comprehension of their health records, especially their doctors' notes.[44–46] In one previous study,[44] we determined that abbreviations cause more misunderstanding than any other barrier, including medical terms and health literacy. In a preliminary study, we assessed the abbreviation content of admissions notes, and determined that abbreviations constitute 30-50% of the words. In the most extreme case, one note began with an entire sentence of acronyms: '50 y/o f w/hx b/l SO pw/ LLQP' (50 year old female with a history of bilateral salpingo-oophorectomy presents with left lower quadrant pain).

Therefore, it is unsurprising that abbreviations frequently cause patients to misunderstand notes. Patients' misunderstanding may reduce the potential benefits of transparency, increase miscommunication, decrease satisfaction, increase doctors' legal liability, and ultimately harm the patient-doctor relationship.[47,48] Because current interventions show extremely limited ability to improve patient's comprehension of abbreviations,[49] any advances in our understanding of the problem and our ability to automatically expand acronyms should have major clinical significance and far-reaching consequences such as better shared decision-making and improved health outcomes.

1.2. Long-Term Goal

Our long-term goal is to develop methods to improve patient comprehension of their health records, focusing specifically on clinical abbreviations. Expanding abbreviations in text is a first step towards fully semantically representing personal health information to all viewers.

1.3. Study Objectives

The overall objective is to establish patient comprehension of abbreviations vs. expansions in clinical text, using several control mechanisms to isolate the main effect of expansion. This assessment is intended to:

(1) characterize the extent of miscommunication due to abbreviations in health records;
(2) evaluate the need for potential solutions such as automated abbreviation expansion.

We *hypothesized* that abbreviation expansion would improve patient comprehension of abbreviations in clinical text. As a secondary analysis, the study will determine associations between comprehension of abbreviations and common characteristics, including education level, socioeconomic status, and health literacy.

1.4. Control of Confounding

The following control mechanisms will be used to isolate the main effect:

- *Control for prior health system exposure:* Patients at the same level of care will be recruited. Specifically, we will recruit primarily heart failure patients that have been recently hospitalized, as this very sick population experiences a high need to quickly comprehend their personal health information.

- *Control for clinical context and prior knowledge of medical jargon:* Patients with the same illness condition will be recruited. Specifically, we have chosen to recruit heart failure patients. Heart failure is a highly prevalent chronic illness that impacts over 6.5 million Americans.[50,51]

- *Control for difficulty of comprehension:* We will include abbreviations of varied difficulty for patients to comprehend, as rated by participating clinicians prior to the design and recruitment of the trial. No abbreviations that clinicians deem overly difficult to comprehend will be included.

- *Control for written context:* We will present abbreviations and expansions in a short paragraph similar to the History of Present Illness (HPI) section of doctors' notes. The participant will read the entire paragraph before comprehension of each abbreviation or expansion is assessed.

2. Study Design

2.1. General

This study is a prospective, two-arm, parallel, individually randomized controlled trial designed to establish patients' objective comprehension (*primary outcome*) of abbreviations vs. expansions in clinical text. Patients will be individually randomized to receive either abbreviations (*control group*) or expansions (*intervention group*).

2.2. Participants

Table 2.1 describes the eligibility criteria for participation. The subject population is 60 diagnosed adult heart failure patients (confirmed by clinical exam, echocardiographic evidence, or cardiologist expert opinion), who receive primary care at one of three institutions: Columbia University Medical Center, Weill Cornell Medical Center, and New York University Langone Health.

We will recruit a purposive sample on age, sex, and race as per the institutions' adult heart failure population demographics on 1 July 2019. Potential participants will be identified by their attending cardiologist and invited to participate by the research coordinator. The attending cardiologists will not be involved in any study procedures, including data collection and randomization, and will not have access to the results except in aggregation.

Table 2.1. Eligibility Criteria

Inclusion Criteria	Exclusion Criteria
Adult (21 years or older)	Healthcare professional (MD, DO, RN, etc.)
Confirmed diagnosis of heart failure	No telephone number or email address
Able to read and speak English	Severe cognitive impairment or clinical diagnosis of dementia
Willing and able to provide informed consent	Major psychiatric illness, including active psychosis
Receives primary care from participating provider	Other illness that would preclude participation

2.3. Baseline Data

All baseline data will be recorded in REDCap. REDCap is a secure, HIPAA-compliant web application for building and managing online surveys and databases. REDCap is specifically geared to support online or offline data capture for research studies and operations.

Table 2.2 describes the baseline data that will be collected. Demographic characteristics included age, gender, race, ethnicity, and preferred language, and socioeconomic characteristics included educational attainment, insurance type, financial resources, and disability status. To estimate health literacy, we use the 3-item Brief Health Literacy Screener.[52]

Table 2.2. Baseline Data

Data Field	Question Text	Coded Responses
mrn_empi	Please enter patient's EMPI number:	Text (10 digit EMPI)
survey_taker	Who is completing this survey?	1 Researcher<br>2 Patient
location	Where was the participant recruited?	1 Inpatient<br>2 Outpatient
institution	Where does the participant receive primary care?	Text (prefilled institution)
age	What is your age?	Text (number, minimum 18, maximum 110)
gender	What gender do you identify as?	1 Male<br>2 Female<br>3 Other
gender_other	Please specify "Other":	Text (required)
race	What race do you identify as?	1 White<br>2 Black<br>3 Asian<br>4 Native American<br>5 Mixed<br>6 Other<br>7 Unsure/prefer not to answer
race_other	Please specify "Other":	Text (required)
ethnicity	What ethnicity do you identify as?	1 Hispanic/Latino<br>2 Not Hispanic/Latino<br>3 Unsure/prefer not to answer
language_spoken	What is your preferred language to speak?	1 English<br>2 Spanish<br>3 Cantonese<br>4 Mandarin<br>5 Russian<br>6 Other
languagespoken_other	Please specify "Other":	Text (required)
language_read	What is your preferred language to read and write?	1 English<br>2 Spanish<br>3 Cantonese<br>4 Mandarin<br>5 Russian<br>6 Other
languageread_other	Please specify "Other":	Text (required)

education	What is the highest level of education that you completed?	1 I have never attended school<br>2 Eighth grade<br>3 Some high school<br>4 High school<br>5 Some college<br>6 Associates degree<br>7 Bachelor's degree<br>8 Master's degree<br>9 Doctoral degree<br>10 Unsure/prefer not to answer
health_insurance	Do you currently have health insurance?	1 Yes<br>2 No<br>3 Not sure
insurance_type	What kind of health insurance do you have?	1 Insurance through employer<br>2 Self purchased insurance<br>3 Medicare<br>4 Medicaid<br>5 Other program<br>6 Not sure what type of insurance
financial_resources	Do you feel you have enough financial resources to make ends meet?	1 More than enough<br>2 Enough<br>3 Not enough
disabled	Do you have a disability?	1 Yes<br>0 No
disability_type	Which of the following describes your disability?	1 Problems with physical mobility<br>2 Problems with hearing<br>3 Problems with seeing<br>4 Other
disability_other	Please specify "Other":	Text (required)
healthlit_1	How often do you have problems learning about your medical condition because of difficulty understanding written information?	1 Never<br>2 Occasionally<br>3 Sometimes<br>4 Often<br>5 Always
healthlit_2	How confident are you with filling out medical forms by yourself?	1 Extremely<br>2 Quite a bit<br>3 Somewhat<br>4 A little bit<br>5 Not at all
healthlit_3	How often do you have someone help you read hospital materials?	1 Never<br>2 Occasionally<br>3 Sometimes<br>4 Often<br>5 Always

2.4. Intervention

This study is designed to compare comprehension of abbreviations vs. expansions in clinical text. We have used the following process to identify abbreviations for inclusion in the randomized trial:

- Using the Clinical Abbreviation Recognition and Disambiguation (CARD) framework,[53] we identified the 20 most common abbreviations found in advanced heart failure notes, excluding those abbreviations judged as highly obscure by participating clinicians (n=6).
- Participating clinicians rated these 20 abbreviations and their corresponding expansions as easy, moderate, or difficult for patients to comprehend. We then choose 10 of the 20 abbreviations of varied difficulty and their corresponding expansions for inclusion in the randomized trial.

Table 2.3 displays the included abbreviations and corresponding clinician ratings.

Table 2.3. Clinician Perceptions of Patient Comprehension of Abbreviations

Clinical Text	Difficulty Score (out of 12)	Estimated Comprehension (% of patients)	Interpretation
**Abbreviations**			
hrs	0	100%	Easy
MD	1	92%	Easy
BP	3	75%	Easy
ED	3	75%	Easy
yo	5	58%	Moderate
pt	5	58%	Moderate
HF	6	50%	Moderate
hx	9	25%	Difficult
HTN	10	17%	Difficult
MI	11	8%	Difficult
**Expansions**			
hours	0	100%	Easy
medical doctor	0	100%	Easy
blood pressure	0	100%	Easy
emergency department	0	100%	Easy
year old	0	100%	Easy
patient	0	100%	Easy
heart failure	0	100%	Easy
history	2	83%	Easy
hypertension	2	83%	Easy
myocardial infarction	9	25%	Difficult

Categories: 75-100% is easy, 50-75% is moderate, and <50% is difficult.

We will present abbreviations or expansions in a short paragraph similar to the History of Present Illness section of doctors' notes **(Figure 2.1)**. This will control for written context and stimulate real-life interpretation of abbreviations or expansions as closely as possible. The paragraph will be short to minimize time burden on participants. Text size will be 20 point font to ensure legibility as per National Institution of Aging recommendations.

(a)

A 44 yo man, former smoker with a hx of gout, HF, and HTN, presents to the ED with chest pain. Pt states that his pain began about 2 hrs ago. BP is 154/96. The MD is concerned for MI.

A 44 year old man, former smoker with a history of gout, heart failure, and hypertension, presents to the emergency department with chest pain. Patient states that his pain began about 2 hours ago. Blood pressure is 154/96. The medical doctor is concerned for myocardial infarction.

(b)

A 44 yo man, former smoker with a hx of gout, HF, and HTN, presents to the ED with chest pain. Pt states that his pain began about 2 hrs ago. BP is 154/96. The MD is concerned for MI.

A 44 year old man, former smoker with a history of gout, heart failure, and hypertension, presents to the emergency department with chest pain. Patient states that his pain began about 2 hours ago. Blood pressure is 154/96. The medical doctor is concerned for myocardial infarction.

Figure 2.1. Paragraphs with abbreviations or expansions

(a) Versions to be displayed to participants during the randomized trial
(b) Versions highlighted according to clinicians' perceptions (easy, moderate, difficult)

2.5. Randomization

Prior to launching recruitment, an independent statistician will randomly assign groups using a computer-generated block randomization algorithm of size 6. Groups will be assigned in a 1:1 ratio.

Two coordinators will enroll participants and collect data. REDCap will conceal each participant's allocation until directly after baseline data collection. Blinding after assignment is not possible due to the obvious nature of the intervention.

2.6. Outcome

The primary outcome will be objective comprehension, assessed using the International Organization for Standardization Method for Testing Comprehension (ISO 9186)[54] as previously adapted for oral administration by an objective observer.[55] ISO 9186 is a well-known standardized protocol for assessing objective comprehension.

Briefly, the coordinator primes participants to the task **(Figure 2.2)** and reads the paragraph aloud. Then, the coordinator asks participants to verbally explain each abbreviation's or expansion's meaning as per ISO 9186. If the response is incomplete or incorrect, the coordinator can only say "tell me more," to prevent them from biasing the response.

We will consider the response correct if the participant can describe the abbreviation or expansion, as determined by the coordinator. The coordinators will discuss the potential scope and depth of adequate responses prior to recruitment to ensure standardization, and any unclear classifications will be discussed by the research team.

Next, I'm going to ask you to read a paragraph.

You may not understand every word in this paragraph and that's ok.

Our goal is to find out what you don't understand, so we can make it more understandable in the future.

[Participant reads paragraph with either abbreviations or expansions]

Now, I'm going to ask you what some of the words mean.

Even if it seems obvious, just say it.

If you don't know, it's okay to say "I don't know."

[Ask about meaning of each abbreviation or expansion]

Figure 2.2. Introductory script to ISO 9186 protocol. Actions depicted in [gray]

2.7. Procedures

Table 2.4 describes the study procedures. In an important update as per version 2.0, participants will be now recruited by phone in addition to in-person at the outpatient clinic or inpatient unit. Recruitment by phone will be used when necessary to comply with local COVID restrictions. We will rigidly adhere to local restrictions to prevent unnecessary coronavirus infections in this vulnerable patient population.

Table 2.4. Study Procedures

Procedure	Description
Prescreening	Patient is prescreened for eligibility and invited to participate by their cardiologist
Consent	Participant provides verbal informed consent (by phone) or written informed consent (in-person)
Baseline	Participant completes baseline questionnaire and health literacy screening
Allocation	Participant assigned to either the abbreviation group (control) or expansion group (intervention)
Assessment	Objective comprehension is assessed as per the ISO 9186 protocol

2.8. Adherence to Guidelines

This study will be performed in accordance with World Medical Association Declaration of Helsinki. The Weill Cornell Medicine Institutional Review Board has approved the trial and all participants will provide informed consent. We will conduct and report this trial in accordance with CONSORT.[56]

Notably, this trial was not classified as a clinical trial under the NIH definition at the time it was funded, so it was not registered prospectively. However, the journal has asked that it be registered retrospectively as they have determined this study to meet the ICMJE definition of a clinical trial. Accordingly, the trial has been retrospectively registered with ClinicalTrials.gov (NCT05297942).

3. Statistical Analysis

3.1. Data Management

The coordinator will enter baseline data and outcome data electronically into REDCap at the time of collection. The coordinators will be trained in the ISO 9186 protocol and a pre-standardized REDCap survey will be used throughout to ensure that data collection is standardized. Data transfer between REDCap and computers for statistical analysis will be HIPAA-compliant and the data will be stored in a secure server environment.

3.2. Sample Size Calculation

Based on the clinician ratings, the estimated comprehension of expansions is 89% and the estimated comprehension of abbreviations is 56%. Therefore, we have anticipated a comprehension rate of 89% for the intervention and 56% for the control group. Our planned sample size of 60 patients will provide 80% power to detect this 33 percentage point difference in our primary outcome at the 5% significance level. We will stop recruitment once 60 participants have completed the trial.

3.3. Main Analysis

We will use a two-sided unequal variances t-test with a significance threshold of $P<0.05$ to analyze differences in overall comprehension (primary outcome) between study groups based on a summary (count) score of the total number of abbreviated or expanded terms comprehended by the patient. Fisher's exact test of independence with a significance threshold of $P<0.05$ will be used to examine differences between abbreviated and expanded versions of each individual term. Uncertainty will be estimated using standard 95% confidence intervals.

We will conduct bivariate analyses to assess whether baseline characteristics differ by group. Nominal variables will be compared using Fisher's exact tests, while ordinal and numerical variables will be compared using Wilcoxon rank-sum tests. Participants will be analyzed according to their original allocation. One goal of the study design is to avoid missing data, and we do not anticipate having missing data. However, in the event of missing data, we will use multiple imputation methods. Analyses will be performed in R Version 3.6.3.[57]

3.4. Subgroup Analysis

We will conduct a subgroup analysis of control group participants to assess potential predictors of objective comprehension of abbreviations. First, correlation (for numerical baseline variables) and t-test or ANOVA (for categorical baseline variables) will be used to determine relationships between individual baseline characteristics and summary (count) scores for the total number of abbreviations comprehended. Based on this preliminary analysis, multiple Poisson regression models may be used to predict summary comprehension scores using selected baseline characteristics (P<0.15 in the bivariate analyses).

4. References

1. Office of the National Coordinator for Health Information Technology. *Percent of Hospitals, By Type, that Possess Certified Health IT, Health IT Quick-Stat #52*. (2018).
2. Adler-Milstein, J. *et al.* Electronic health record adoption in us hospitals: Progress continues, but challenges persist. *Health Aff.* **34**, 2174–2180 (2015).
3. Adler-Milstein, J. *et al.* Electronic health record adoption in US hospitals: the emergence of a digital 'advanced use' divide. *J. Am. Med. Inform. Assoc.* **24**, 1142–1148 (2017).
4. Adler-Milstein, J. *et al.* More Than Half of US Hospitals Have At Least A Basic EHR, But Stage 2 Criteria Remain Challenging For Most. *Health Aff.* **33**, 1664–1671 (2014).
5. Delbanco, T. *et al.* Open notes: doctors and patients signing on. *Ann. Intern. Med.* **153**, 121–5 (2010).
6. Delbanco, T. *et al.* Inviting patients to read their doctors' notes: a quasi-experimental study and a look ahead. *Ann. Intern. Med.* **157**, 461–70 (2012).
7. Walker, J. *et al.* Inviting patients to read their doctors' notes: patients and doctors look ahead: patient and physician surveys. *Ann. Intern. Med.* **155**, 811–9 (2011).
8. American Hospital Association. *Individuals' Ability to Electronically Access Their Hospital Medical Records, Perform Key Tasks is Growing*. (2016).
9. Irizarry, T. *et al.* Patient Portals as a Tool for Health Care Engagement: A Mixed-Method Study of Older Adults With Varying Levels of Health Literacy and Prior Patient Portal Use. *J. Med. Internet Res.* **19**, e99 (2017).
10. Irizarry, T., DeVito Dabbs, A. & Curran, C. R. Patient Portals and Patient Engagement: A State of the Science Review. *J. Med. Internet Res.* **17**, e148 (2015).
11. Nielsen-Bohlman, L. *Health literacy: a prescription to end confusion*. (2004).
12. Sarkar, U., Karter, A. J. & Liu, J. Y. The Literacy Divide: Health Literacy and the Use of an Internet- Based Patient Portal in an Integrated Health System—Results from the Diabetes Study of Northern California (DISTANCE). *J Heal. Commun* **15**, 183–196 (2010).
13. Goel, M. S. *et al.* Patient reported barriers to enrolling in a patient portal. *J. Am. Med. Informatics Assoc.* **18**, i8–i12 (2011).
14. Weingart, S. N. *et al.* Lessons from a patient partnership intervention to prevent adverse drug events. *Int. J. Qual. Heal. Care* **16**, 499–507 (2004).
15. Weingart, S. N. *et al.* Medication safety messages for patients via the web portal: The MedCheck intervention. *Int. J. Med. Inform.* **77**, 161–168 (2008).
16. Heyworth, L. *et al.* Engaging patients in medication reconciliation via a patient portal following hospital discharge. *J. Am. Med. Informatics Assoc.* **21**, e157–e162 (2014).
17. Schnipper, J. L. *et al.* Effects of an online personal health record on medication accuracy and safety: a cluster-randomized trial. *J. Am. Med. Informatics Assoc.* **19**, 728–734 (2012).
18. Dullabh, P. M., Sondheimer, N. K., Katsh, E. & Evans, M. A. How patients can improve the accuracy of their medical records. *EGEMS (Washington, DC)* **2**, 1080 (2014).
19. Staroselsky, M. *et al.* Improving electronic health record (EHR) accuracy and increasing compliance with health maintenance clinical guidelines through patient access and input. *Int. J. Med. Inform.* **75**, 693–700 (2006).
20. Caligtan, C. A., Carroll, D. L., Hurley, A. C., Gersh-Zaremski, R. & Dykes, P. C. Bedside information technology to support patient-centered care. *Int. J. Med. Inform.* **81**, 442–451 (2012).
21. Dalal, A. K. *et al.* A web-based, patient-centered toolkit to engage patients and caregivers in the acute care setting: A preliminary evaluation. *J. Am. Med. Informatics Assoc.* **23**, 80–87 (2016).
22. Stade, D. & Dykes, P. Nursing Leadership in Development and Implementation of a Patient-Centered Plan of Care

Toolkit in the Acute Care Setting. *CIN Comput. Informatics, Nurs.* **33**, 90–92 (2015).
23. Maher, M. *et al.* A Novel Health Information Technology Communication System to Increase Caregiver Activation in the Context of Hospital-Based Pediatric Hematopoietic Cell Transplantation: A Pilot Study. *JMIR Res. Protoc.* **4**, e119 (2015).
24. Maher, M. *et al.* User-Centered Design Groups to Engage Patients and Caregivers with a Personalized Health Information Technology Tool. *Biol. Blood Marrow Transplant.* **22**, 349–358 (2016).
25. Dykes, P. C. *et al.* Building and testing a patient-centric electronic bedside communication center. *J. Gerontol. Nurs.* **39**, 15–9 (2013).
26. Kruse, C. S., Bolton, K. & Freriks, G. The effect of patient portals on quality outcomes and its implications to meaningful use: a systematic review. *J. Med. Internet Res.* **17**, e44 (2015).
27. Mold, F. *et al.* Patients' online access to their electronic health records and linked online services: A systematic review in primary care. *Br. J. Gen. Pract.* **65**, e141–e151 (2015).
28. Bitton, A., Poku, M. & Bates, D. Policy context and considerations for patient engagement with health information technology. in *Information Technology for Patient Empowerment in Healthcare* (eds. Grando, M., Rozenblum, R. & Bates, D.) 75–90. (Walter de Gruyter Inc., 2015).
29. 2014 Edition EHR Certification Criteria Grid Mapped to Meaningful Use Stage 2. Available at: https://www.healthit.gov/sites/default/files/2014editionehrcertificationcriteria_mustage2.pdf. (Accessed: 12th March 2016)
30. American Hospital Association. *Individuals' Ability to Electronically Access Their Hospital Medical Records, Perform Key Tasks is Growing*. (2016).
31. HealthIT.gov National Learning Consortium. *How to Optimize Patient Portals for Patient Engagement and Meet Meaningful Use Requirements*. (2013).
32. OpenNotes.org. (2020). Available at: https://www.opennotes.org/.
33. Wolff, J. L. *et al.* Inviting patients and care partners to read doctors' notes: OpenNotes and shared access to electronic medical records. *J. Am. Med. Inform. Assoc.* **157**, 461–470 (2016).
34. Bell, S. K. *et al.* A patient feedback reporting tool for OpenNotes: Implications for patient-clinician safety and quality partnerships. *BMJ Qual. Saf.* **26**, 312–322 (2017).
35. Nazi, K. M., Turvey, C. L., Klein, D. M., Hogan, T. P. & Woods, S. S. VA OpenNotes: exploring the experiences of early patient adopters with access to clinical notes. *J. Am. Med. Informatics Assoc.* **22**, 380–389 (2014).
36. Leveille, S. G. *et al.* Evaluating the impact of patients' online access to doctors' visit notes: designing and executing the OpenNotes project. *BMC Med. Inform. Decis. Mak.* **12**, 32 (2012).
37. Bell, S. K. *et al.* When doctors share visit notes with patients: a study of patient and doctor perceptions of documentation errors, safety opportunities and the patient-doctor relationship. *BMJ Qual. Saf.* **26**, 262–270 (2017).
38. Gerard, M., Fossa, A., Folcarelli, P. H., Walker, J. & Bell, S. K. What patients value about reading visit notes: A qualitative inquiry of patient experiences with their health information. *J. Med. Internet Res.* **19**, (2017).
39. Goldzweig, C. L. Pushing the Envelope of Electronic Patient Portals to Engage Patients in Their Care. *Ann. Intern. Med.* **157**, 525 (2012).
40. Ammenwerth, E., Schnell-Inderst, P. & Hoerbst, A. The Impact of Electronic Patient Portals on Patient Care: A Systematic Review of Controlled Trials. *J. Med. Internet Res.* **14**, e162 (2012).
41. Zeng-Treitler, Q., Goryachev, S., Kim, H., Keselman, A. & Rosendale, D. Making texts in electronic health records comprehensible to consumers: a prototype translator. *AMIA Annu. Symp. Proc.* 846–50 (2007). doi:amia-0846-s2007 [pii]
42. Polepalli Ramesh, B., Houston, T., Brandt, C., Fang, H. & Yu, H. Improving patients' electronic health record comprehension with NoteAid. *Stud. Health Technol. Inform.* **192**, 714–8 (2013).
43. Schillinger, D. *et al.* The Next Frontier in Communication and the ECLIPPSE Study: Bridging the Linguistic Divide in Secure Messaging. *J. Diabetes Res.* **2017**, (2017).
44. Grossman, L. V, Masterson Creber, R., Restaino, S. & Vawdrey, D. K. Sharing Clinical Notes with Hospitalized Patients via an Acute Care Portal. *AMIA Annu. Symp. Proc.* **2017**, 800–809 (2017).
45. Grossman, L. V *et al.* Implementation of acute care patient portals: recommendations on utility and use from six early adopters. *J. Am. Med. Inform. Assoc.* **25**, 370–379 (2018).
46. Keselman, A. *et al.* Towards consumer-friendly PHRs: patients' experience with reviewing their health records. *AMIA Annu. Symp. Proc.* **2007**, 399–403 (2007).

47. Manson, A. Language concordance as a determinant of patient compliance and emergency room use in patients with asthma. *Med. Care* **26**, 1119–1128 (1988).
48. Waitzkin, H. Doctor-patient communication. Clinical implications of social scientific research. *JAMA* **252**, 2441–6 (1984).
49. Ramesh, B. P., Houston, T., Brandt, C., Fang, H. & Yu, H. Improving patients' electronic health record comprehension with NoteAid. in *Studies in Health Technology and Informatics* **192**, 714–718 (2013).
50. Ambrosy, A. P. *et al.* The global health and economic burden of hospitalizations for heart failure: Lessons learned from hospitalized heart failure registries. *J. Am. Coll. Cardiol.* **63**, 1123–1133 (2014).
51. Benjamin, E. J. *et al.* Heart Disease and Stroke Statistics-2017 Update: A Report From the American Heart Association. *Circulation* **135**, e146–e603 (2017).
52. Chew, L. D., Bradley, K. A. & Boyko, E. J. Brief questions to identify patients with inadequate health literacy. *Fam. Med.* **36**, 588–94 (2004).
53. Wu, Y. *et al.* A long journey to short abbreviations: developing an open-source framework for clinical abbreviation recognition and disambiguation (CARD). *J. Am. Med. Inform. Assoc.* **24**, e79–e86 (2017).
54. International Organization for Standardization. *International Standard ISO 9186-1:2014(E) Methods for Testing Comprehensibility*. (2014).
55. Arcia, A. *et al.* Modifications to the ISO 9186 Method for Testing Comprehension: Successes and Lessons Learned. *2019 IEEE Work. Vis. Anal. Healthc.* (2019).
56. Schulz, K. F., Altman, D. G., Moher, D. & CONSORT Group. CONSORT 2010 statement: updated guidelines for reporting parallel group randomized trials. *Ann. Intern. Med.* **152**, 726–32 (2010).
57. R Core Team. *R: A Language and Environment for Statistical Computing*. (R Foundation for Statistical Computing, 2015).
