# Supplementary material for: Effect of Expansion of Abbreviations and Acronyms on Patient Comprehension of Their Health Records: A Randomized Clinical Trial
Source: JAMA Netw Open. 2022 May 13;5(5):e2212320. doi: 10.1001/jamanetworkopen.2022.12320 (PMC9107024; doi:10.1001/jamanetworkopen.2022.12320)
Supplement: Supplement 2. — eFigure. CONSORT Flow Diagram [file jamanetwopen-e2212320-s002.pdf]

Supplemental Online Content

Grossman Liu L, Russell D, Reading Turchioe M, Myers AC, Vawdrey DK, Masterson Creber RM. Effect of expansion of abbreviations and acronyms on patient comprehension of their health records: a randomized clinical trial. *JAMA Netw Open.* 2022;5(5):e2212320. doi:10.1001/jamanetworkopen.2022.12320

eFigure. CONSORT Flow Diagram

This supplemental material has been provided by the authors to give readers additional information about their work.

© 2022 Grossman Liu L et al. *JAMA Network Open.*

eFigure. CONSORT Flow Diagram

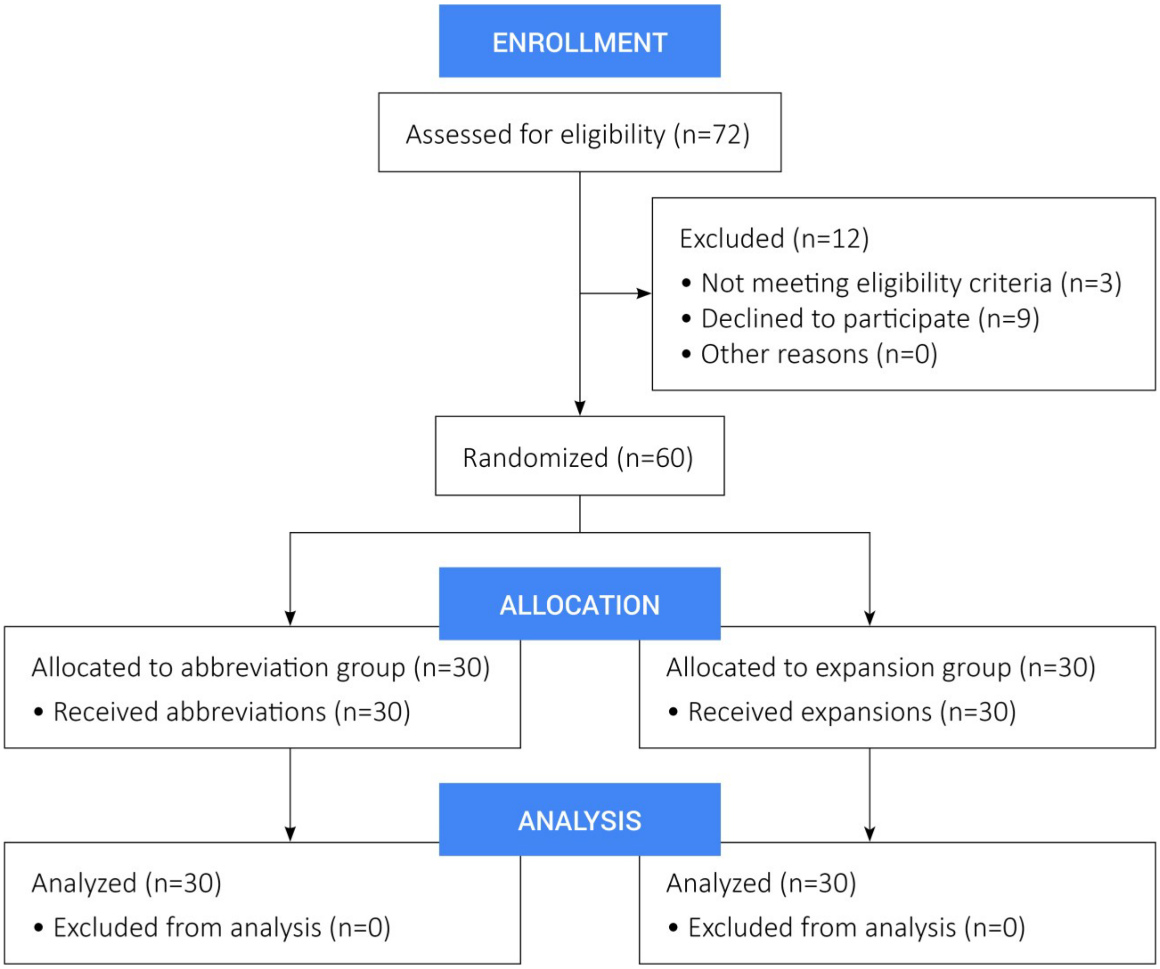

© 2022 Grossman Liu L et al. *JAMA Network Open.*
