# Supplementary material for: Effect of Expansion of Abbreviations and Acronyms on Patient Comprehension of Their Health Records: A Randomized Clinical Trial
Source: JAMA Netw Open. 2022 May 13;5(5):e2212320. doi: 10.1001/jamanetworkopen.2022.12320 (PMC9107024; doi:10.1001/jamanetworkopen.2022.12320)
Supplement: Supplement 3. — Data Sharing Statement [file jamanetwopen-e2212320-s00.pdf]

# Data Sharing Statement

Grossman Liu. Effect of Expansion of Abbreviations and Acronyms on Patient Comprehension of Their Health Records. *JAMA Netw Open*. Published May 13, 2022.
doi:10.1001/jamanetworkopen.2022.12320

**Data**
**Data available:** Yes
**Data types:** Participant data with identifiers, Data dictionary
**How to access data:** lvg2104@cumc.columbia.edu
**When available:** With publication

**Supporting Documents**
**Document types:** None

**Additional Information**
**Who can access the data:** The final locked dataset and any information that exceeds a journal's page limits for publication will be shared with other investigators or healthcare organizations upon request and without cost. Before the request is fulfilled, the investigator or organization must sign a data-sharing agreement to demonstrate and provide for: (1) a commitment to using the information only for research or clinical purposes and not to identify any individual participant, (2) a commitment to securing the information using appropriate computer technology, (3) a commitment to not share the information outside the immediate research or clinical setting, and (4) a commitment to erasing the data after the analyses are completed.
**Types of analyses:** research or clinical purposes
**Mechanisms of data availability:** with a signed data access agreement
**Any additional restrictions:** NA
